# Integration of Sm_2_Co_17_ Micromagnets in a Ferromagnetic Multipolar Microrotor to Enhance MEMS and Micromotor Performance

**DOI:** 10.3390/mi15070875

**Published:** 2024-07-01

**Authors:** Efren Diez-Jimenez, Alberto Bollero, Ignacio Valiente-Blanco, Ester M. Palmero, Miguel Fernandez-Munoz, Diego Lopez-Pascual, Gabriel Villalba-Alumbreros

**Affiliations:** 1 Mechanical Engineering Area, Universidad de Alcalá, 28801 Alcalá de Henares, Spain; i.valiente@uah.es (I.V.-B.); miguel.fm@uah.es (M.F.-M.); gabriel.villalba@uah.es (G.V.-A.); 2 Group of Permanent Magnets and Applications, IMDEA Nanociencia, 28049 Madrid, Spain; alberto.bollero@de.bosch.com (A.B.); ester.palmero@imdea.org (E.M.P.); 3 Electrical Engineering Area, Universidad de Alcalá, 28801 Alcalá de Henares, Spain; d.lopezp@uah.es

**Keywords:** multipolar rotor, MEMS, micromagnets, microassembly, micromotors

## Abstract

MEMS and micromotors may benefit from the increasing complexity of rotors by integrating a larger number of magnetic dipoles. In this article, a new microassembly and bonding process to integrate multiple Sm_2_Co_17_ micromagnets in a ferromagnetic core is presented. We experimentally demonstrate the feasibility of a multipolar micrometric magnetic rotor with 11 magnetic dipoles made of N35 Sm_2_Co_17_ micromagnets (length below 250 μm and thickness of 65 μm), integrated on a ferromagnetic core. We explain the micromanufacturing methods and the multistep microassembly process. The core is manufactured on ferromagnetic alloy Fe_49_Co_49_V_2_ and has an external diameter of 800 μm and a thickness of 200 μm. Magnetic and geometric measurements show good geometric fitting and planarity. The manufactured microrotor also shows good agreement among the magnetic measurements and the magnetic simulations which means that there is no magnetic degradation of the permanent magnet during the manufacturing and assembly process. This technique enables new design possibilities to significantly increase the performance of micromotors or MEMS.

## 1. Introduction

Microelectromechanical systems (MEMS) have become one of the pillars of microelectronics development. Motors [1,2,3,4], clutches/brakes [5,6,7,8], micro-magnetic gears [9,10], vibrational energy harvesters/dampers [11], and other micro-electromagnetic devices [12] have inspired growing interest in recent years. The miniaturization of motors and MEMS potentially opens new frontiers in optical measurement tools [13], electronics for data storage [14,15], optical positioning systems [16], robots for small cavity exploration [17], and manipulators for the manufacturing and assembly of micro-actuators and transducers [18]. Of special interest, micromotors and rotary actuators systems can be used to build up complex microtools for internal medical applications, as in optical gastroscopy [19], colonoscopies [20], intravascular imaging [21], laparoscopic surgery [22], or localized drug delivery [23]. High-torque thin electromagnetic micromotors are critical components that require specific development [24].

These new tools will demand high-torque actuation systems to provide strong grabbing and moving capacity. Actuators typically include a gearhead that multiplies the torque [25,26], but for MEMS and micromotors, it is much more challenging to implement such components due to increased complexity and a greater number of parts [27,28,29]. Specific developments related to micromotors on the microscale are scarcely found in the literature. Details of a stepping micromotor design based on a ferrofluid bearing were published in 2018 [30]. This design follows a permanent magnet (PM)-based micromotor design and it is oriented for accurate angle-positioning of the micromirrors. An additional example was published by Waldschik et al. [31], consisting of a flat epitaxial growth micromotor with a diameter of 8 mm. It included helical coils with about ten turns wrapped around each NiFe pole shoe. The torque density of these two previous examples barely reached 0.025 kNm/m^3^. A third micromotor can be found in reference [32], delivering up to 0.04 kNm/m^3^. These designs provide very low torque density since they do not include high-performance micromagnets and the number of poles in the rotor is just 1 or 2. Another interesting example is found in reference [33], where a microactuator of 2 mm in diameter and 5 mm in length achieves 0.2 kNm/m^3^. In comparison, the torque density of macroscale highly efficient motors is in the range of 0.6 to 8 kNm/m^3^. This means that most performant MEMS and micromotors provide, in the best-case scenario, one order of magnitude lower torque density and power density than their macroscopic equivalents. In this sense, improving the complexity of the rotary parts by reaching a larger number of magnetic poles (multipolar magnetic structures) and achieving better magnetic properties is an important target to raise MEMS and micromotor performance.

Multipolar magnetic structures can be created by pulse magnetization on magnets [34,35,36], using a magnetizing fixture with copper wire. If a high pulse current passes through the fixture, it produces a magnetizing field that is strong enough to permanently magnetize the micro-magnet. Special consideration must be taken when operating at low temperatures because the magnetic properties of the materials may vary significantly [37,38]. However, for micro-magnets, this approach cannot be easily realized because the fixture must be smaller than the micro-magnets themselves. Thus, different approaches have been used for micro-magnet multipole magnetization. Previous developments have demonstrated the creation of multipoles in hard magnetic films [39], using a combination of fixed electrical conductors and soft magnetizing heads to imprint periodic north/south magnetic poles. Several works can be found in the literature that demonstrate multipolar magnetization (even on the micro-scale) of NdFeB permanent magnets [40,41]. One common technique is based on locally heating up a previously magnetized magnet. A back-mounted permanent magnet, with alternative polarity, helps to revert the magnetization of the heated parts of the magnets when heating stops. In these cases, permanent damage is observed in such experimental developments after magnetization.

Other techniques are based on thermomagnetic patterning, using it to make patterns with lateral dimensions down to ~70 μm, but this is only in the relative surface of the layer (1-μm deep) [42]. Additionally, a technique based on the use of a single laser-machined soft magnetic head to selectively reverse the magnetization direction in a hard magnetic layer was developed39. The main limitation of the previously described techniques is that the inversion of magnetic polarization is only superficially achieved, with the magnetic product remaining in the polarized volumes thus being smaller than the potentially achievable one.

A different technique has been proposed for magnetization patterning in macroscale magnets [43]. This technique generates magnetization patterns by locally magnetizing the bulk magnet. This technique has been successfully used for macroscale magnets, providing a magnetic pixel size as small as 4 mm [44] and a thickness greater than 3 mm, which is still large for MEMS and micromotor applications.

In this work, we propose a new multipolar ferromagnetic rotor design that simplifies the magnetization process. We experimentally demonstrate for the first time the feasibility of a multipolar micrometric magnetic rotor with 11 magnetic dipoles made of N35 Sm_2_Co_17_ (referred to as SmCo in the following sections) micromagnets, integrated in a ferromagnetic core with no magnetic degradation. This ferromagnetic core was manufactured on Fe_49_Co_49_V_2_ (referred to as FeCo in the following sections), with an external diameter of 800 μm and a thickness of 200 μm. Complete morphological and magnetic characterization was also performed, and the results were correlated.

## 2. MEMS and Micromotor Design

**Figure 1:**
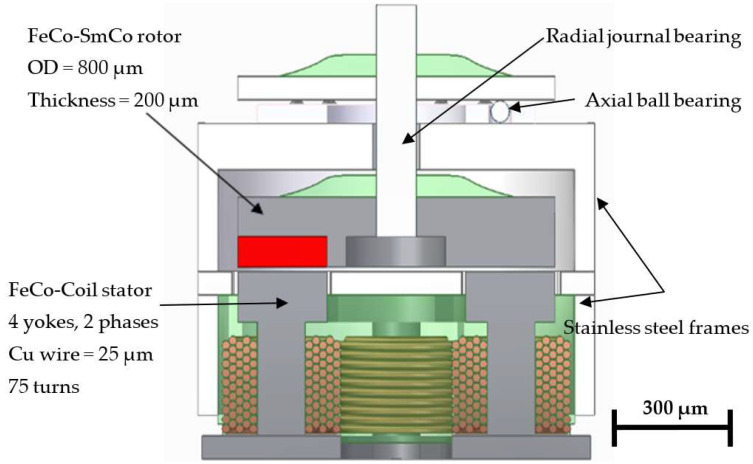
Mechanical and magnetic components of the high-performance design of the proposed micromotor. Motor outer diameter (OD) = 1000 μm, motor height = 1000 μm. Air gap = 10 μm.

Multipolar arrangements allow the design of complex micromachines that can reach higher performance levels in terms of torque density and efficiency. A new axial flux stepper Vernier stepper motor is proposed to increase the torque density of MEMS and the micromotor. This topology includes four stator yokes activated in two phases. It also includes a magnetic flux modulator that maximizes the resulting torque while reducing the cogging torque. This motor also requires a multipolar magnetic rotor where the number of dipoles matches the number of teeth on the magnetic flux modulator. In total, 11 magnetic dipoles are included in the rotor, while the flux modulator has 12 soft ferromagnetic modulating teeth. This generates a magnetic gearing effect with a 1:11 ratio that allows it to overcome the excessive cogging torque caused by scale effects. The mechanical design also includes an axial ball micro bearing [45] and a radial plan bearing to keep the rotor in place. The non-magnetic parts should be manufactured in titanium or a non-magnetic stainless steel like AISI-316. The complete design is shown and is further described in Figure 1.

**Figure 2:**
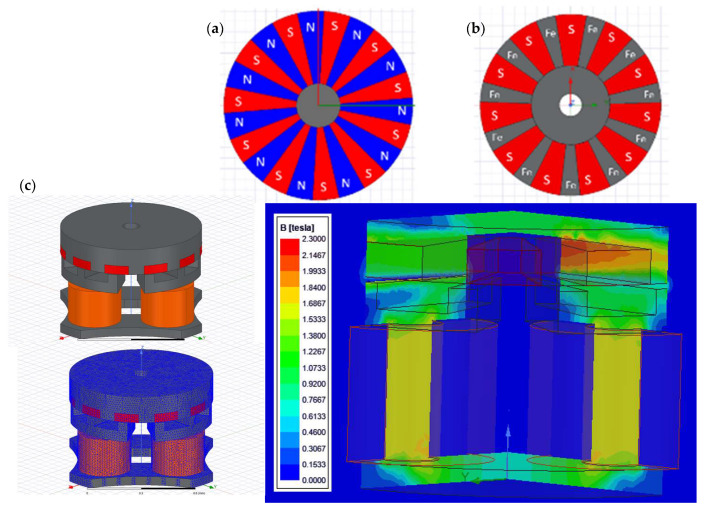
(**a**) Rotor with multipolar alternating magnetization directions (north–south): OD = 800 μm, (**b**) multipolar single magnetization direction: OD = 800 μm, and (**c**) FEM model, meshed, with magnetic field results after simulation.

An electromagnetic simulation using ANSYS Electronics has been performed to determine the expected torque of the proposed design. Two different designs for the rotor were considered, using a multipolar alternant magnetization direction and multipolar single magnetization direction (Figure 2a,b). The first option is a typical north–south alternant polarity configuration (Figure 2a). The manufacturing process of this first option is simpler, as only a single permanent magnet piece is needed. However, obtaining a multipolar arrangement of this size on a single magnet is very difficult, because of the complexity of creating a multipolar copper fixture for multipolar magnetization with such a small diameter. It would also require unachievably high current densities to enable local magnetization in such an alternating situation. Therefore, although it is simpler to manufacture and assemble a single magnet, this option was discarded due to the unfeasible magnetization process. Other alternatives for multipolar magnetization also have severe limitations that would prevent achieving the maximum torque levels.

**Figure 3:**
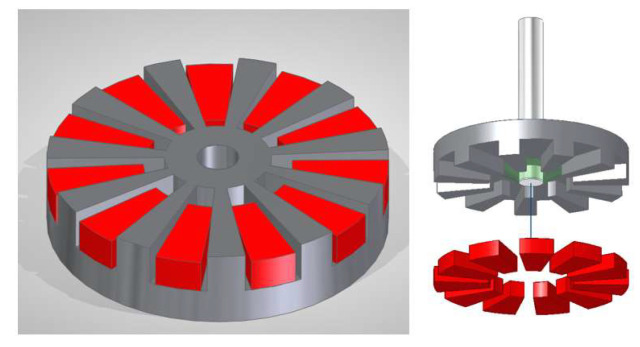
Option B rotor design (gray: soft ferromagnetic FeCo; red: SmCo micromagnets). Outer diameter = 800 µm, total height = 200 µm. Horizontal clearances between micromagnet and yoke = 20 µm.

The second option is a combination of soft ferromagnetic teeth with individual hard permanent magnets integrated between the magnetic teeth, as shown in Figure 2b. This second option is more complex to manufacture as the ferromagnetic rotor requires a grooving process, and hard permanent magnets have to be manufactured in smaller micrometric sizes; in addition, this second option requires an integration process to position the magnets in the ferromagnetic yoke. In contrast, the magnetization process of this option is much more straightforward, as a single magnetizing pulse can be applied using a standard magnetizing fixture. Therefore, this second option is the only one that turned out to be feasible in all its required steps from a practical point of view, while assuring the highest level of magnetic properties in the resulting rotor, and, thus, the highest torque and performance of the micromotor, Figure 3.

The simulation was conducted using a magnetostatic 3D analysis of the complete motor, as shown in Figure 2c. The materials used for the ferromagnetic FeCo (Hc = 50 A/m and Jsat = 2.3 T) rotor and the micromagnets (SmCo Recoma 35E were sourced from Arnold Magnetics, with Br = 1.18 T and Hcb = 870 kA/m). The mesh is shown in Figure 2c. Boundary conditions were set as a zero tangential field in a surrounding volume that was three times larger than the motor envelope.

The performance and torque simulation results for both options are given in Table 1 and are shown in Figure 2c. Both options present very similar performance; therefore, the decision to select one option or the other is based on the manufacturability and magnetic quality of the prototype. The mechanical design includes 20 µm of horizontal clearance between the micromagnet and yoke to ensure a good clearance fit. There is also a difference of 10 µm in the design between the height of the micromagnet and the depth of the yoke grooves. This ensures that the top surface of the FeCo ferromagnetic yoke will always be the highest point so it can be used as the reference surface to set the rotor–stator airgap. The choice of samarium as a permanent magnet material is due to two fundamental reasons: one is its resistance to corrosion and the other is its high or higher resistance to temperature. It is expected that when the motor is running, the temperature of the assembly will be working internally at around 45–50 °C. The torque performance that the motor can offer varies by around 0.25%/°C, decreasing as the temperature increases.

In this work, we present the development of an integration method with Sm_2_Co_17_ micromagnets in a ferromagnetic multipolar microrotor to enhance the performance of MEMS and micromotors. The experimental results of the manufacture and assembly of the single magnetization direction option will be described later.

## 3. Microfabrication of Parts

The rotor requires the manufacturing of several parts: 11 SmCo micromagnets and 1 FeCo alloy ferromagnetic rotor yoke, with 11 pockets to hold the micromagnets.

### 3.1. Sm_2_Co_17_ Micromagnets

A novel damage-free ultrashort pulsed laser machining process was used to manufacture the complex shapes of the Sm_2_Co_17_ micromagnets [46]. The micromagnets were manufactured using an ultrashort pulsed hydro laser micromilling process. The laser milling system used a commercial laser source (TRUMPF TrueMicro 2030, TRUMPF, Farmington, NM, USA) operating at a wavelength of 1030 nm, with a pulse width of 400 fs, a maximum pulse energy of 100 µJ, and 20 W of power, using a liquid-cooled part chuck and with the cutting speed set at 30 µm/s.

The choice of Sm-Co provides clear advantages when compared to NdFeB material for an application of this type: the higher corrosion resistance of Sm-Co makes the avoidance of a protective coating layer possible in this application, which may be critical when using NdFeB with a micrometer size below 250 µm (in terms of control over coating thickness when using conventional commercial coating technologies or expense when moving to alternative techniques such as sputtering or atomic layer deposition), in addition to a significantly higher resistance against demagnetization with increasing temperature (which, when combined with a temperature-controlled cutting process, diminishes enormously the local demagnetization risk that might occur during cutting). The laser process used here allows for further miniaturization of the resulting micromagnets, achieving micromagnets with longer dimensions below 250 µm and a height of 65 µm. This can be achieved because the micromagnets are submerged in a coolant fluid, which avoids temperature escalation during the cutting process and, thus, avoids the partial demagnetization of the magnets that might likely result from the application of a high processing temperature. Complex segment shapes, made in high-quality Sm_2_Co_17_ material with good accuracy, can be achieved with this method. It has been demonstrated elsewhere that no permanent degradation of magnetic properties appears after laser machining [46].

**Figure 4:**
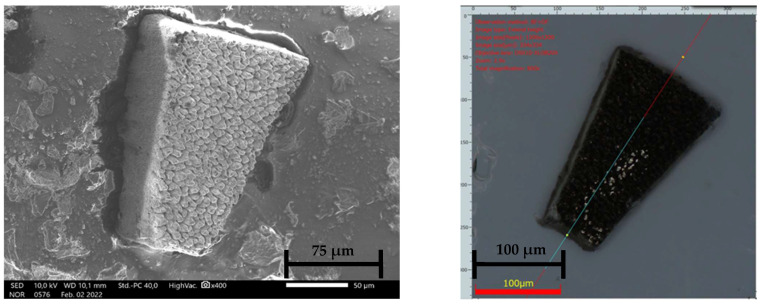
SmCo micromagnets, manufactured using the hydro laser micromilling process.

The machining process consists of three steps. The first step involves reducing the thickness of the raw material from 110 μm to 65 μm through laser surface milling, Figure 4. The next step is rinsing, which is necessary to remove any remaining particles from the disc, which is now at the target thickness. The final step is the precise cutting of the microsegments using an automatic grooving process.

### 3.2. Ferromagnetic Rotor Yoke, Made in Fe_49_Co_49_V_2_

**Figure 5:**
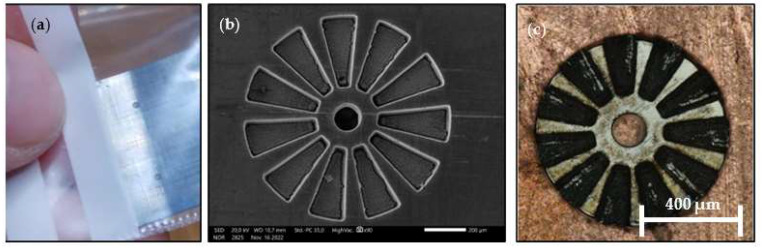
(**a**) FeCo pockets being made in the laminate, (**b**) scanning electron microscopy (SEM) image of the resulting grooves after laser micromilling, and (**c**) the resulting ferromagnetic rotor after microCNC milling.

This part requires a different machining method. Initially, femtosecond laser micromachining was used to create the pockets that would host the magnets, as shown in Figure 5 (top views). Then, microCNC was used to cut out the outer diameter of the pockets, following the micromanufacturing technique described in [47].

## 4. Integration Process

The process of integrating the micromagnets into the ferromagnetic yoke follows similar steps to those that could be proposed for a macro-scale prototype. The steps are as follows: (i) choose non-magnetized micromagnets; (ii) place them in the definitive positions in the ferromagnetic rotor; (iii) apply a magnetizing pulse (this step generates an attractive force between the magnets and the yoke that keeps the micromagnets in place); and (iv) seal and protect the micromagnets once in position with an adhesive. However, the difficulty and novelty of the integration process lies in the very small size of each of the components, which is in the micrometer range. This has made it necessary to develop specific micro-tools for each of the steps to be performed. The following subsections describe in detail each of the processes, with their peculiarities and novelties. All the processes were completed inside a clean-room vertical laminar air flow cabinet ISO 5 Tesltar Aeolus V.

### 4.1. Pick and Release System for Microparts

**Figure 6:**
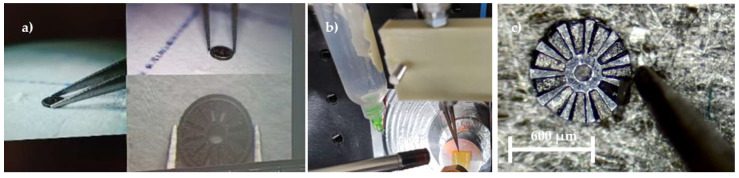
(**a**) Metallic micro tweezers gripping the rotor, (**b**) negative pressure needle (in green), and (**c**) tip for final positioning of the micromagnet against the ferromagnetic rotor.

The pick and release process, i.e., picking up, moving, and releasing the micro magnets and the ferromagnetic yoke, has been performed in three different ways. In the first, and most simple way, the ferromagnetic rotor yoke was picked up using metallic tweezers, the opening and closing of which were controlled by a linear actuator Z825B from Thorlabs with a minimum step of 0.5 μm. Therefore, the gripping motion can be completed smoothly (Figure 6a).

For the micromagnets, which were more fragile, using the micro grippers was not an option since they would damage them. Accordingly, a negative-pressure pick-up and release microsystem was developed. It included a metallic microneedle with a 25 μm hole at the tip and a small suction pump. The negative pressure that was generated held the magnets against the needle tip. The last method for the final positioning of the micromagnets consists simply of using a wire with a diameter of 25 μm. The time taken to place a micromagnet in the right position was about 1–2 min; therefore, in around 15–20 min, the rotor can be assembled using manual control and automatic motion stages.

### 4.2. Micropositioners

**Figure 7:**
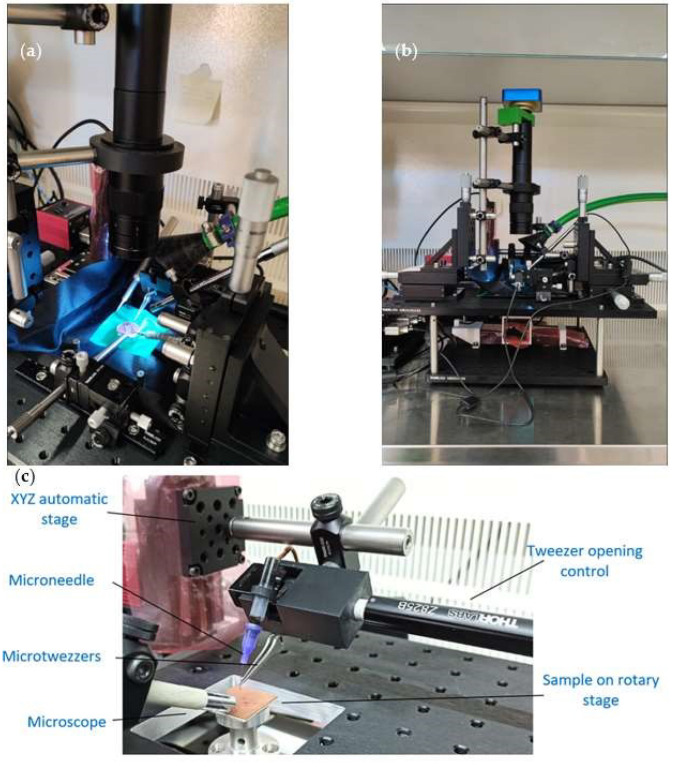
(**a**) Positioning system, showing the automatic stages for the micropart displacement and the manual stage of focusing the microscopes, (**b**) lateral view of the system inside the clean-room cabinet, and (**c**) detailed view showing the linear actuator that controls the microtweezers’ opening and closing.

The micropositioning system is a compilation, using different precision components to handle and assemble the microelements of the motor, complete adhesive microdroplet deposition, and perform magnetic field measurements (Figure 7). This system is composed of two automatic XY translation stages, plus an additional rotation stage placed on a lower bench and a Z-travel translation stage on an upper bench. This gives four degrees of freedom to the baseplate where the assembly is performed. All the stages can be not only computer-controlled but also manually controlled using a gamepad. The lower-level stages are visible through a central access point in the upper-level bench. This upper bench has a large surface on which to place the required tools for each operation, like microtweezers and microneedles. The system also includes 1 centered vertical high-resolution microscope and several thin USB microscopes, which permits a view of the process from 3–4 different viewpoints simultaneously (Figure 6a). Thanks to the automatic translation stages, great precision has been achieved and the different sequences can be executed. This system allows different subsystems that are easy to assemble and disassemble, so this configuration is versatile and very useful, providing that microscopes are always present.

The integration process starts with the placement of all the components of the rotor and the whole set of micro magnets on a substrate base, preferably made of copper, with a ground connection to avoid and/or decrease any electrostatic charges. This initial component placement is performed manually with manual micro hand grippers. Once the components are located within a close working area of less than 25 by 25 mm, the micro positioning system is used for the final approach of the components to their final position. It is important to discharge all elements electrostatically with ground connections, as well as to make sure beforehand with a magnetometer that there are no significant magnetic fields in the vicinity. As the components have a negligible weight, any magnetic field can carry them further away and displace them, and they will then disappear completely from the field of view.

### 4.3. Magnetization

As intended in the design, the magnetization process is simple and straightforward. This is a great advantage as any other multipolar alternating magnetization technique might not achieve full and complete magnetization; thus, it will exhibit reduced performance. Once the micromagnets were placed in the groove, a small PTFE laminate top was placed over them and taped in place to prevent the micromagnets from escaping from the grooves in the next processing step. Then, the whole rotor was placed on a plastic stopper and inserted into the magnetizing solenoid inner core. The magnetizer NCD-1100/2-24T from E-magnet can generate up to 5.64 T, a sufficiently large magnetic field that can achieve the complete magnetization of SmCo magnets. The rotor was inserted in the magnetizing fixture and three pulses were activated.

The design of the rotor ensures that when the micromagnets start to be magnetized, all are aligned in the same direction. As long as they remain magnetized, they are attracted to the bottom FeCo ferromagnetic rotor yoke, keeping them in position with a very strong attraction force. After magnetization, the micromagnets are completely joined to the rotor yoke.

### 4.4. Gluing Microdrop System

**Figure 8:**
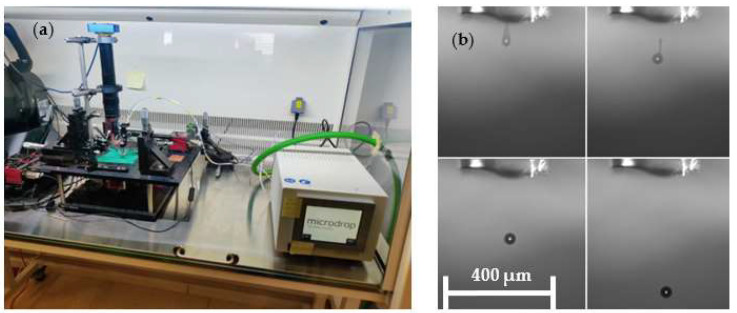
(**a**) Adhesive micro drop dispenser for the positioning stages, and (**b**) adhesive drops generated by the piezoelectric actuator, comprising spheres of 40 μm in diameter.

The positioning system may also include an automatic adhesive micro dispensing system (Figure 8a). This system consists of the controller of an injection system that is deformed by a piezoelectric actuator and that will release adhesive drops in a gradual and controlled manner (MD-K-140 from Microdrop GmbH, Norderstedt, Germany). The drops can be as small as 40 μm in diameter (quasi-spherical shape), as shown in Figure 8b. This subsystem has been integrated into the automatic assembly and gluing system in such a way that the adhesive drops can be deposited in a controlled manner, with a precision of at least ±25 μm, in the desired location. This system requires the use of adhesives of low viscosity that can be cured by an external action such as ultraviolet light or temperature.

The last step of the integration process consists of the coating and gluing of the micromagnets with a small drop of ultraviolet-curing adhesive (NOA 61 from Norland Products, Jamesburg, NJ, USA). When the adhesive approaches the groove area, the surface tension makes it cross the small space between the height of the micromagnet and the height of the groove, which was intentionally designed to achieve this. The height difference is about 10–15 μm.

The drop generates a meniscus between the side wall of the ferromagnetic yoke and the top wall of the micromagnet. Then, this process is applied to every one of the grooves, and the unit is subsequently kept under high-frequency ultraviolet light for 20 s. After this step, the magnets are perfectly sealed, protected, and placed in the corresponding grooves.

## 5. Integration Results

The resulting rotor has been analyzed from two main perspectives according to its final use: geometrically and magnetically.

### 5.1. Geometric Characterization

**Figure 9:**
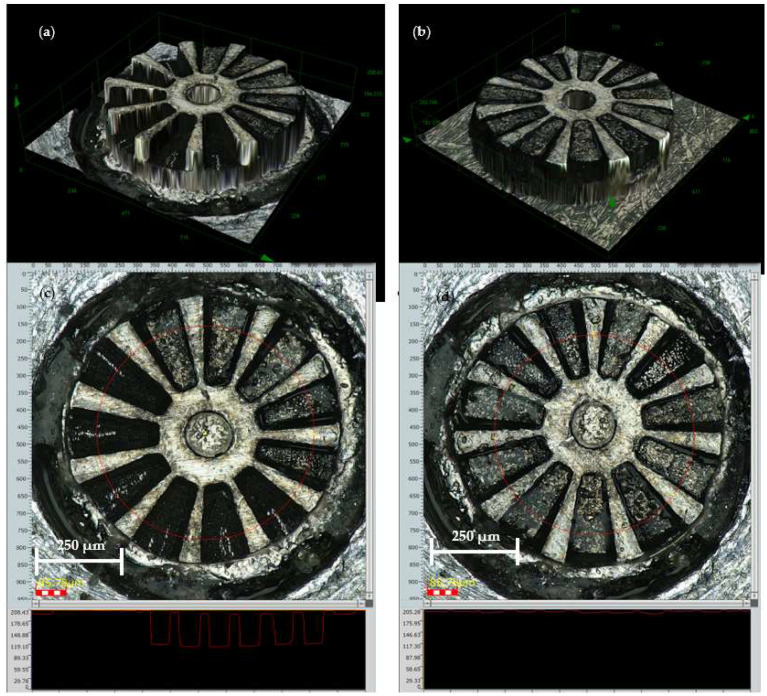
(**a**) A 3D view of the rotor after the assembly of five micromagnets, (**b**) 3D view of the rotor with all the magnets, (**c**) profile of the rotor in the intermediate state showing the six remaining pockets, and (**d**) profile of the rotor once completed.

The geometric characterization of the integrated micromagnets in the rotor was performed using a DSX1000 Digital Microscope from Olympus, Tokyo, Japan. This microscope can make accurate measurements using a telecentric optical system. It can take measurements on the focal plane within ± 1 µm, as well as generate 3D images of the objects, producing profilometric measurements in both lines (1D) and surfaces (2D). Figure 9 shows several images of the resulting sample at 2 different moments in the process, first with 5 micromagnets inserted in 5 of the 11 available grooves, and then after complete integration and magnetization.

The rotor now includes all the micromagnets in the right position, with some radial clearance of 20 µm in size between the outer diameter of the micromagnet and the outer diameter of the rotor. This clearance was intentionally designed to ensure a clearance fit between the two parts and to ensure that they would fit together easily. It is estimated that this clearance could be reduced to a level of 5 μm.

**Figure 10:**
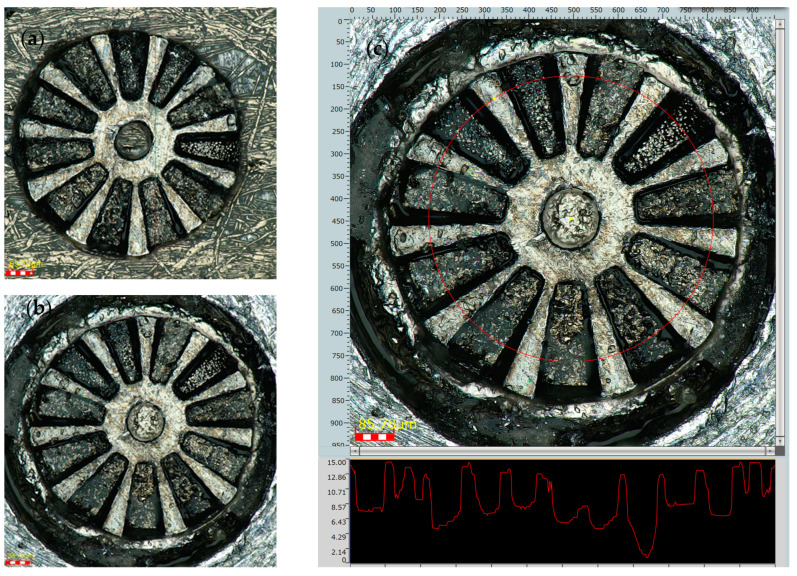
(**a**) Top view of the rotor, (**b**) second image of the results on a different substrate, and (**c**) profile measurement along a circumference including the FeCo and the SmCo magnets.

Figure 10c shows a circumferential measurement of the height profile at an average diameter of the rotor, which is marked on the figure itself. It is evident that there is a step in height difference of 7 μm approximately, corresponding to each micromagnet.

Figure 10a,b shows that all the magnets (making a total of 11) are in perfect condition, with no chips or deterioration. The outer diameter of the rotor is measured in 800 µm. The top surface roughness is smaller than 3 µm.

From these measurements, it can be concluded that the manufacture and integration of the magnets is correct and in accordance with the design, with dimensional position differences of less than 7%. The established clearances of 20 μm are more than sufficient for feasible integration and this could be reduced even further.

### 5.2. Magnetic Characterization

Magnetic characterization was performed using a micro hall effect sensor HG 0711 from the AKM company (Tokyo, Japan), located above the rotor. The exact height between the hall sensor element position and the rotor top surface is 150 μm. The distance between the hall sensor element and the end of the epoxy encapsulation of the sensor was previously calibrated using a two-track PCB, current, and simulation. Once the rotor-hall element was calibrated, a horizontal sweep was performed in the horizontal XY plane in 50 µm steps, using the automatic linear stages described in Section 4. At each position, the vertical magnetic field in the Z direction that was generated by the rotor was measured.

**Figure 11:**
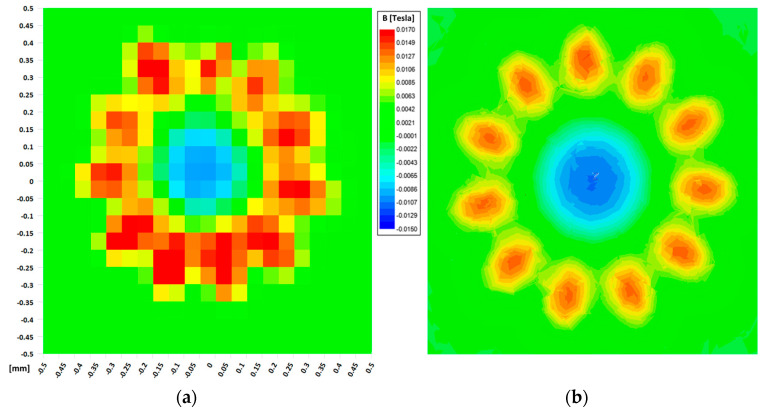
((**a**)—left) Experimental measurements of the vertical magnetic field at a distance of 150 µm, and ((**b**)—right) simulation results at a distance of 150 μm (both graphs refer to the same scale).

The results of these measurements are shown in Figure 11a. It is clear that the magnetic field is higher in the location just above the micromagnets. This level is around 17 mT. There are 10 peaks in the magnetic field that are clearly visible, corresponding to each of the micromagnets. A magnetic field in the opposite sense can be observed at the center. A magnetic field beyond 800 μm in diameter decays rapidly.

These experimental measurements were then compared to the simulation results. A finite element magnetostatic simulation of the rotor in isolation was performed using a ferromagnetic FeCo (Hc = 50 A/m and Jsat = 2.3 T) rotor and the micromagnets, assuming their best magnetic properties (SmCo Recoma 35E from Arnold Magnetics (Rochester, NY, USA) where Br = 1.18 T and Hcb = 870 kA/m).

The simulation results in Figure 11b show the calculated field in the vertical component, at a height of 150 µm above the reference rotor surface.

It can clearly be seen that the simulation and experimental values and field distribution are in good agreement, there being differences of less than 5% of the absolute value.

It can, therefore, be concluded that the process does not deteriorate the magnetic properties of either the micromagnets or the yoke. With this fabrication and integration method, a multipolar micrometric rotor with highly magnetic properties can be constructed in an efficient way.

Future development of this method includes the integration of a larger number of dipoles, applications using NdFeB, which has greater magnetic properties, and the reduction of clearance tolerances.

## 6. Conclusions

This study demonstrates experimentally the feasibility of a multipolar micrometric magnetic rotor with 11 magnetic dipoles made of N35 SmCo micromagnets (with a length below 250 μm and a thickness of 65 µm) integrated into a ferromagnetic core. This ferromagnetic core was manufactured using FeCo, with an external diameter of 800 μm and a thickness of 200 μm. A new microassembly and bonding process was developed to integrate the SmCo micromagnets into a ferromagnetic core. In this paper, we show the micromanufacturing methods and the multistep microassembly process. Magnetic and geometric measurements demonstrate good geometric fitting and flatness. The results also show good agreement between the magnetic measurements and the magnetic simulations, which means that there is no magnetic degradation. This experimental method opens up new design possibilities to significantly increase the performance of electromagnetic micromachines like micromotors or MEMS.

## Figures and Tables

**Table 1:** Performance comparison of the two magnetization options for the rotor.

	Multipolar Alternating Magnetization Direction	Multipolar Single Magnetization Direction
Torque (μNm)	2.03	2.15
Current (A)	3.8	3.8
Torque density (kNm/m^3^)	7.98	8.45
Cogging torque (%)	5.6	4
Axial load bearing (mN)	68	70

## Data Availability

The information and all data given in this article are accessible under request to authors.
